# Impact of Pharmacy Based Travel Medicine with the Evolution of Pharmacy Practice in the UK

**DOI:** 10.3390/pharmacy6030064

**Published:** 2018-07-09

**Authors:** Derek Evans

**Affiliations:** FRPharmS, FRGS, MFTM RCPS, Independent Prescriber, 58 The Nurseries, Langstone, Wales NP18 2NT, UK; d.p.evans@btinternet.com

**Keywords:** travel medicine, health, pharmacist, pharmacy, vaccinations, prescribing and education

## Abstract

Background: Pharmacy has utilised the changes in legislation since 2000 to increase the range and supply function of services such as travel health to travellers. With the number of travellers leaving the UK and trying new destinations there is an increasing need for more travel health provision. Working models: The models of supply of a travel health service vary according to the size of the corporate body. The large multiples can offer assessment via a specialist nurse or doctor service and then supply through the pharmacy. Others will undertake an onsite risk assessment and supply through the pharmacist. The sole Internet suppliers of medication have been reviewed and the assessment standards questioned following survey and inspection. Education: There is no dedicated pharmacist-training programme in advanced level travel health. As a consequence one academic institution allows pharmacists to train on a multidisciplinary course to obtain an academic membership. With training for travel health not being mandatory for any travel health supply function the concern is raised with standards of care. Future: There is a consultation paper on the removal of travel vaccines from NHS supply due to be decided in the future. If these vaccines are removed then they will provide a greater demand on pharmacy services. Discussion: The starting of a travel health service can be made without any additional training and remains unregulated, giving cause for concern to the supply made to the traveller. Conclusions: Pharmacies in the UK offer a range of options for supplying travel health services; however these need to be with improved mandatory training and supply.

## 1. Introduction

This is a review of the development of the practice of pharmacy in the UK developing from the legal changes that occurred in the 2000s to include modern working models and specialist eduction that is available to pharmacists.

In the UK, prior to 2000 the role of the pharmacist was that of the traditional supply function against the supply of a prescription and the sale of over the counter products (OTC). Within the UK the legislation is defined in 3 legal categories of medicines, prescription only (POM)—only supplied against a doctors or dentists prescription (private and National Health Service (NHS)); pharmacy only medicines (PM)—only supplied from a licensed pharmacy in the presence of a pharmacist and OTC products. At this time there was no legislation that allowed pharmacists to provide routine or travel vaccinations or to supply POMs without a relevant prescription.

With changes to both the legal supply of POMs and the increasing requirement to use pharmacist’s clinical skills then evolvement of influenza vaccination was introduced. This led onto the consideration of travel health health services from a pharmacy, by a pharmacist, to become established.

The changes to a nationally funded travel health service remain under scrutiny and with increasing numbers of patients travelling abroad annually (+5%) and +27% intending to to travel a country they have never visited before [1] the NHS is reviewing the current funding of these services.

## 2. Legislative Changes

**Figure 1:**
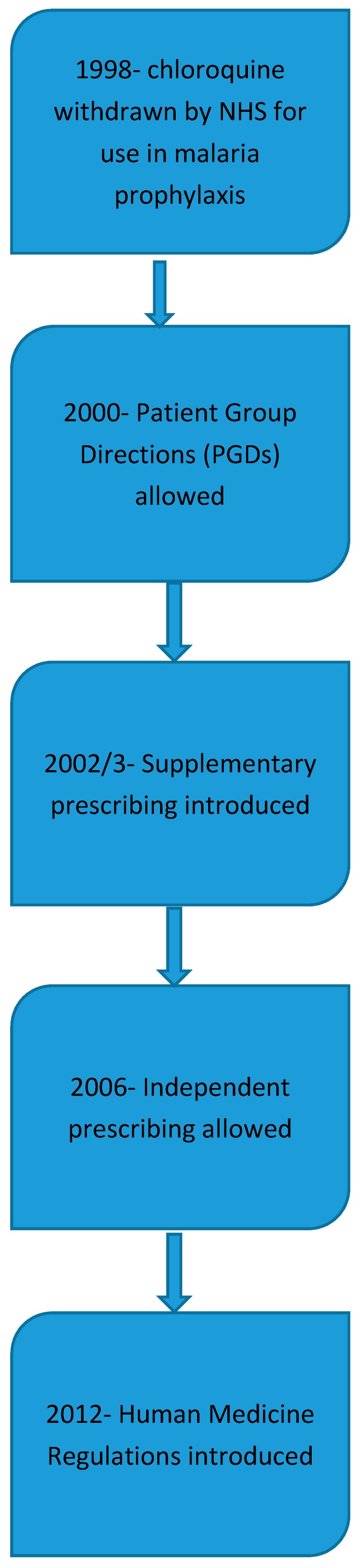
Key legislative changes allowing pharmacists to practice travel medicine.

In 1998 the NHS reviewed the medicines that where be allowed to be supplied on a prescription. This review included the removal of chloroquine for malaria prophylaxis and the change to PM status allowing it to be purchased from pharmacies, (Figure 1). The NHS continues to supply free of charge to all travellers’ vaccines against hepatitis A, typhoid, diphtheria/tetanus/polio and cholera from surgery that is contracted to provide vaccinations. All other vaccinations remained on a private supply along with the antimalarials.

In 2000 following lobbying from the Royal College of Nurses (RCN) for group protocols to supply national immunisation services, the law was changed to allow the supply of medication by healthcare professionals using Patient Group Directions [2].

At the same time-work was underway to allow a new category of prescribers to be formed. This became known as supplementary prescribing, which was originally given to practice and district nurses working alongside a doctor to reduce the workload [3]. A supplementary prescriber was a healthcare professional who had the authority to supply a range of previously agreed drugs according to an agreed clinical management plan.

With the increase in clinical knowledge and skills being taught and applied to other healthcare professions the role of independent prescriber was created in 2006, which included pharmacists [4]. This role allowed a pharmacist, with a formal qualification in independent prescribing to be able to write diagnose, treat and write prescriptions. The ethical area of competence is unregulated and such prescribers a can legally write a prescription that may include schedule 2 Controlled Drugs [5].

A major change in the law occurred in 2012 with the introduction of the Human Medicines Regulations [6]. This allowed the widening of the range of medication that could be supplied under a PGD allowing more services to be created. Additional NHS regulations introduced at the same time allowed the concept of a new style of pharmacy, that of the at distance or “postal service” pharmacies, where a pharmacy could make an online sale or supply of a prescription without face to face contact with the patient. Alongside these regulations came the new standards of pharmacy premises which included the minimum design standards for every pharmacy to have a their own consultation room to provide services.

With the UK Government reviewing the impact of annual influenza absences and the pressure for new roles to be allowed to pharmacy the first pharmacy flu vaccination services were introduced in 2007 as a private service utilising PGDs. This continued until 2015 when it was extended and included as a pharmacy NHS funded service.

Alongside the provision of seasonal influenza vaccinations other vaccination services supplied by PGDs and Pharmacist Independent Prescriber (PIPs) evolved such as travel vaccinations and the supply of antimalarials for prophylaxis. With the austerity measures imposed on public spending private clinical services were considered as a part solution to maintaining financial solvency.

In late 2017 the PHE was tasked with a consultation between professionals to consider if the total withdrawal of all vaccines for pre-travel should be removed from the NHS [7] and made private supply only.

The significance of the changes to practice working at a faster rate than legislative changes has led to the introduction of support networks for pharmacists providing travel health service using telemedicine or at distance triage services completed by a nurse or doctor [8].

## 3. Models of Working Practices

Within the pharmacy population in the UK, 49.2% of pharmacies are owned by groups and termed large multiples. Examples of these include Boots, Lloyds, Well, Rowlands and the supermarket groups of Asda, Morrisons and Tesco. Other smaller independents provide 12.4% and the remainder is made up small chains (<3 outlets) and independents 38.4% of the market [9].

Access to travel health or medicine services is varied and one type of model includes a risk assessment being completed externally by a doctor, nurse or pharmacist and then the vaccination or anti-malarial supply being authorised by either a private prescription or directed to a pharmacist through a Patient Specific Direction (PSD). (A PSD is a formal arrangement, which allows a doctor or independent prescriber to direct the supply of vaccination through another healthcare professional to a patient).

Other pharmacies, allow an online booking service to be made with one of its selected stores (not all stores are included) for a risk assessment lasting up to 40 min. The clinical support and backup is provided by a nurse led service to which the pharmacist can refer. The pharmacist can administer the vaccinations and supply the medication at the appointment.

The UK legislation differs at this point regarding the regulation of the standards of practice between solely organised pharmacist clinics and those by other healthcare professions. The regulating body in England, the Care Quality Commission (CQC) [10] regulates the standards to be applied in nurse or doctor led clinics; whilst the General Pharmaceutical Council (GPhC) regulates the standards in pharmacies. In 2017 the joint regulators investigated online prescribing to patients. The report highlighted significant areas of failure between some online prescribers and those in a patient-facing scenario [11]. A previous study evaluating the supply of atovaquone/proguanil through online prescribing highlighted that potential questions were no addressed such as previous ADR to the drugs (59%) or the length of stay in the malarial area (50%) [12].

A small independent survey study in 2018 concluded that a pharmacy travel health service was well accepted by patients and met their needs, providing a value for money service [13].

## 4. Travel Health Education

With the supply of POM medication made by pharmacists either under a PGD or as an Independent Prescriber the professional expectation is that the standard of training to use these preparations should be of the a similar standard at an advanced level. Within the UK there is no legal requirement to have received any formal advanced level training before using a PGD and whilst an Independent Prescriber qualifies in a defined area of competence, they are legally allowed to prescribe any medicinal product outside of their competence, including controlled drugs.

To ensure that basic immunisation is practiced correctly the GPhC in alliance with the Royal College of Nursing and Public Health England have published a document that lists the national minimum standards and core curriculum for immunisation training for registered healthcare practitioners [14].

For those pharmacists who do elect to undertake extended training there are ranges of courses in travel health that are shared with other professions allowing an equality between practitioners. Examples of this can be found at the centres of excellence of in London, Liverpool and Glasgow. Details of the courses available to pharmacists are seen in Table 1, an overview suggests that pharmacy professional courses are of a basic level and many of the advanced level courses are restricted to registered doctors and nurses only.

Additionally an external representative body, the British Global Travel Health Association (BGTHA) has produced its own e-learning programme that is currently awaiting accreditation.

## 5. Future

The Association of British Travel Agents travel trends report [1] indicates that there is a growth in early bookings for 2018 holidays and people are travelling to destinations not visited before. In the summer of 2017 the UK government announced a review of cost controls throughout areas of the NHS. Amongst this was a proposal to remove all vaccines currently provided free of charge for travel (hepatitis A, typhoid, combined tetanus/diphtheria/polio, and cholera) from NHS supply. The NHS has since requested a feasibility study to be completed by Public Health England [7] on the complete removal of these vaccines and the subsequent impact on public health services. The findings are awaiting publication.

## 6. Discussion

Within the UK the changes in national legislation have provided wider and more extensive powers for pharmacists to supply POM medication. The term travel health is undefined and yet to be recognised by the General Medical Council as a medical speciality. The consequence is that a pharmacist can initiate a travel health service and the levels of service being offered can vary from the supply of antimalarial medication to specialists who have completed an extensive level of training. To become competent in travel health, pharmacist practitioners should consider the need to complete an independent prescribers course, a recognised formal qualification in travel health and membership to a medical Royal College. This has been accomplished by a small number of pharmacist practitioners, however the practice of travel health without all of the additional skill sets, relies on the pharmacist to understand the limits of their competency when assessing and providing a clinical service.

As highlighted before, the supply function of POMs can be made using a PGD, however no mandatory training on the use is required to use these. The other supply function is using an independent prescriber that trains in a specific area of competency but are legally allowed to prescribe any POM. A consequence of this is that pharmacist independent prescribers can supply travel health medication without being specifically trained in the specialist clinical area.

The professional guidelines advise that independent prescribers should only practice within their competency and that PGDs are only used following specific training. Due to the complex nature of travel health a review is required of the supply provision and training and that should include arrangements for referral of complex patient cases to specialist pharmacists. The need of specialist, mandatory training is supported by the Faculty of Travel Medicine who have published a statement indicating that there is no licensing or checks on the level of care and that travellers are at risk [15].

By comparison in Alberta, Canada pharmacist provided travel health services are supplied by suitably trained pharmacists with basic life support skills and prescribing skills. The additional requirement is the completion of a formally recognised qualification in travel health, such as the Certificate of Knowledge of the International Society of Travel Medicine [16].

## 7. Conclusions

Travel health provision through UK community pharmacies is well advanced due to changes in national legislation. The supply of POM medications using PGD and independent prescriber services allows any pharmacist to privately supply travel health medication; however the minimum skill base to provide these services remains undefined and not legally required. To ensure the ongoing safety of travellers then the UK licensing authorities need to consider working with the specialist education providers to define minimum standards of competence for pharmacists. The formal training of advanced level services available to pharmacists is supplied through a single awarding institution, with other institutions selectively offering to medical practitioners and nurses only. To match the demand of pharmacist level travel health services more formally certified post-graduate courses in travel medicine would need to be made available. The future would indicate that with more people travelling there would be an increased demand on travel health services in the UK.

## Figures and Tables

**Table 1:** Travel health training and education available to pharmacists in UK.

Institution	Travel Medicine Post-Graduate Course Available to Pharmacist	Formal Professional Accreditation
Faculty of Travel Medicine (FTM) of the Royal College of Physicians and Surgeons (Glasgow)	Diploma in Travel Medicine (12–15 months)	Membership of Faculty of Travel Medicine of RCPS
	Foundation in Travel Medicine (6 months) https://rcpsg.ac.uk/travel-medicine/education	None
London School of Hygiene and Tropical Medicine	None-professional diplomas only available to physicians based in London and nurses/midwives (3–12 months) https://www.lshtm.ac.uk/study/courses/professional-development/professional-diplomas	None
	Pharmacists can have access to a short course (4 days) https://www.lshtm.ac.uk/study/courses/short-courses/travel-medicine	None
Liverpool School of Tropical Medicine	Travel Vaccination- Principles and Practice (5 weeks)	None
	Malaria prevention in Travel Health (3 weeks) http://www.lstmed.ac.uk/study/courses	None
Centre for Pharmacy Postgraduate Education	Travel health- understanding and supporting travellers’ wellbeing https://www.cppe.ac.uk/programmes/l/travel-e-02/	Evidence for Royal Pharmaceutical Society Faculty framework https://www.rpharms.com/professional-development/faculty/about-the-faculty
National Pharmacy Association	Travel PGDs https://www.npa.co.uk/training/patient-group-directions/travel-pgd/	None
British Global Travel Health Association (BGTHA)	ABC of travel health https://www.abcoftravelhealth.com	None
National Travel Health Network and Centre (NaTHNaC)	Yellow training and clinic registration https://nathnacyfzone.org.uk	Accreditation to provide Yellow Fever vaccination and registration of clinic
